# 

**Published:** 1915-03

**Authors:** 


### Deaths.

Dr. W. T. Childress died February 2, 1915, at his home in Terrell.

Dr. C. F. Yeager, a practicing physician of Mineral Wells for the past thirty-four years, died January 20, 1915.

Dr. David Morris Thurston, one of the most prominent physicians of Southwest Texas, died in a San Antonio sanitarium January 25, 1915.

Dr. Fred Helton, a well known physician of Stillwell, died January 29, 1915.

